# Bowel inflammation in cancer patients: the microbiome, antibiotics and interleukin-9

**DOI:** 10.1038/s41416-020-01030-0

**Published:** 2020-08-26

**Authors:** Niels Halama

**Affiliations:** 1 Department of Translational Immunotherapy, German Cancer Research Center (DKFZ), Heidelberg, Germany; 2 HI-TRON Helmholtz Institute for Translational Oncology, Mainz, Germany

**Keywords:** Cancer microenvironment, Translational research

## Abstract

Microbiome composition can impact disease courses and also immunotherapy outcomes in solid tumours. It is still unclear how the microbiome might impact treatments in oncology, but also how modulation via antibiotics might interfere. Elegant work now identified interleukin-9 and dysbiosis as relevant factors, providing some answers for these questions.

## Main

The microbiome has attracted increasing scientific attention in the last 5 years.^1–4^ In light of the better understanding of the composition of what constitutes the microbiome in the gut and in other organs,^5,6^ one central question is still out in the open: how much influence the composition of the microbiome exerts on organ function and can we modulate this? There are elegant studies in animal models, indicating, among other things, that there is a direct relationship between the composition of the microbiome in the gut and immunotherapy outcome.^3,5,7–9^

Looking beyond animal models into the clinical situation, the picture becomes much fuzzier. Do we need to treat patients with antibiotics^10–12^ (or other biome-modulating agents) to induce treatment responses in oncology? Do we need to refrain from giving antibiotics to patients for fear of abrogating anti-tumour responses? Published data show poorer treatment responses in patients with antibiotics,^13–15^ but this is not a surprise for an oncologist: patients who need antibiotic treatment are clearly worse in their overall outlook compared with those who do not need antibiotics. And even one step beyond this: “what constitutes a ‘normal’ microbiome”?^16^ Is there a holy grail of beneficial bugs living in the guts of super-responders? These are questions being investigated currently and we have to wait for more insights before we can move to action in the clinic.

**Fig. 1:**
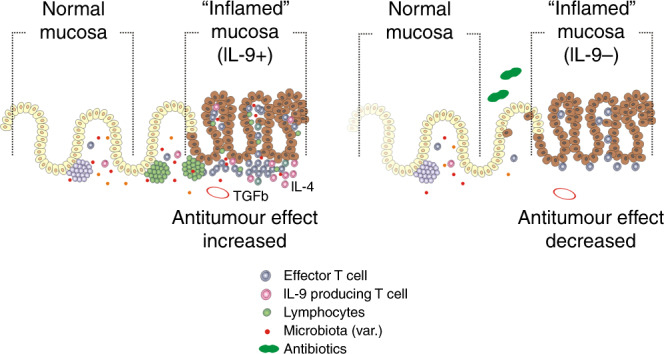
Differential effects for IL-9 in the mucosa. Schematic summary of the differential effects of microbiome composition (i.e. differing microbial composition and dysbiosis shown on the right panel) on the generation of interleukin-9-producing T cells.

In light of these pressing questions, one aspect now has been addressed in the manuscript from Almeida et al.^17^ This elegant work now brought up interleukin-9 (IL-9) as an induced parameter by specific microbial communities. IL-9 is a well-known immunologically pleiotropic molecule.^18,19^ In inflammatory bowel disease, gut-residing T cells produce high amounts of IL-9. In model systems of colitis, IL-9-producing T cells critically interfered with an intact barrier function of the intestinal epithelium by modulating cellular proliferation and tight junction control. Inhibiting IL-9 ameliorated the inflammation and severity of inflammatory bowel disease. As for cancer diseases, this indicates, in light of the new data from Almeida et al.,^17^ that there could be a link to gut inflammation, which enforces anti-tumoural effects through barrier-breaching and bacterial product presence beyond the gut. It is an interesting hypothesis that comes from this: is there a beneficial inflammatory signature that is mediated by IL-9 and resembles a state of colitis? Almeida et al.^17^ found that the host microbiota enhances in vivo T-cell-derived secretion of IL-9, thereby limiting cancer outgrowth (see Fig. 1). So maybe we have to rethink the current clinical concepts for gut inflammation and antibiotic use. We need to understand better inflammatory states especially for IL-9^20^ and not rush too easily into combating all forms of inflammation. There is more clinical and biological data from translational studies needed to clarify this situation.

## Data Availability

Not applicable
